# Trait anxiety mediates stress-related psychopathology after cardiac surgery and ICU stay

**DOI:** 10.1186/cc14630

**Published:** 2015-03-16

**Authors:** L Kok, M Sep, D Veldhuijzen, S Cornelisse, A Nierich, J Van der Maaten, P Rosseel, J Hofland, J Dieleman, C Vinkers, L Peelen, M Joëls, D Van Dijk, M Hillegers

**Affiliations:** 1 University Medical Center Utrecht, the Netherlands; 2 Leiden University, the Netherlands; 3 Isala Clinics, Zwolle, the Netherlands; 4 University Medical Center Groningen, the Netherlands; 5 Amphia Hospital, Breda, the Netherlands; 6 Erasmus Medical Center, Rotterdam, the Netherlands

## Introduction

ICU survivors are at risk for post-traumatic stress disorder (PTSD) and depression. The development of psychopathology depends partially on stable personality factors such as trait anxiety. Among ICU patients a high level of trait anxiety is relatively common and associated with intrusions, a symptom of PTSD. Independently, childhood trauma and stress exposure throughout life have been associated with depression. In cardiac surgery patients admitted to the ICU postoperatively, the effect of trait anxiety on the relationship between cumulative life stress and stress-related psychopathology remains unknown. Therefore we aimed to assess the mediating or moderating role of trait anxiety in this at-risk patient population.

## Methods

In this multicenter follow-up study of the Dexamethasone for Cardiac Surgery (DECS) trial, validated self-report questionnaires were sent 1.5 to 4 years after cardiac surgery and ICU treatment to assess symptoms of PTSD and depression, in relation to cumulative life stress (that is, childhood trauma, major stressful life events) and trait anxiety as determinants of psychopathology. Data were available for 1,125 out of 1,244 (90.4%) eligible participants. Mediating and moderating analyses were performed with multivariable linear regression to assess the effect of trait anxiety. Subgroup analyses were performed for both sexes.

## Results

Trait anxiety partially mediates the relationship between cumulative life stress and PTSD (β-value reduction from 0.325 to 0.068; *P *= 0.000 to *P *= 0.003) and fully mediates the association between cumulative life stress and depression (β-value reduction from 0.282 to 0.015; *P *= 0.000 to *P *= 0.507). Trait anxiety was not a moderating factor between cumulative life stress and psychopathology. Full mediation of trait anxiety was found in female patients (*n *= 247), whereas only partial mediation was seen in male patients (*n *= 878) with regard to PTSD symptoms. As for depression, full mediation was present in both female and male patients.

## Conclusion

In cardiac ICU patients, trait anxiety mediates the influence of cumulative life stress on the occurrence of PTSD and depression symptoms. Further prospective research is necessary to establish these factors as reliable measures for the early identification of ICU patients at risk for stress-related psychopathology.

